# Cardiac contractility is a key factor in determining pulse pressure and its peripheral amplification

**DOI:** 10.3389/fcvm.2023.1197842

**Published:** 2023-06-23

**Authors:** Francesco Piccioli, Ye Li, Alessandro Valiani, Valerio Caleffi, Phil Chowienczyk, Jordi Alastruey

**Affiliations:** ^1^ Department of Engineering, University of Ferrara, Ferrara, Italy; ^2^ King’s College London British Heart Foundation Centre, Department of Clinical Pharmacology, St Thomas’ Hospital, London, United Kingdom; ^3^ Division of Imaging Sciences and Biomedical Engineering, King’s College London, St. Thomas’ Hospital, London, United Kingdom

**Keywords:** aortic flow, hypertension, pulse pressure, cardiac contractility, arterial compliance

## Abstract

**Background:**

Arterial stiffening and peripheral wave reflections have been considered the major determinants of raised pulse pressure (PP) and isolated systolic hypertension, but the importance of cardiac contractility and ventricular ejection dynamics is also recognised.

**Methods:**

We examined the contributions of arterial compliance and ventricular contractility to variations in aortic flow and increased central (cPP) and peripheral (pPP) pulse pressure, and PP amplification (PPa) in normotensive subjects during pharmacological modulation of physiology, in hypertensive subjects, and *in silico* using a cardiovascular model accounting for ventricular–aortic coupling. Reflections at the aortic root and from downstream vessels were quantified using emission and reflection coefficients, respectively.

**Results:**

cPP was strongly associated with contractility and compliance, whereas pPP and PPa were strongly associated with contractility. Increased contractility by inotropic stimulation increased peak aortic flow (323.9 ± 52.8 vs. 389.1 ± 65.1 ml/s), and the rate of increase (3193.6 ± 793.0 vs. 4848.3 ± 450.4 ml/s^2^) in aortic flow, leading to larger cPP (36.1 ± 8.8 vs. 59.0 ± 10.8 mmHg), pPP (56.9 ± 13.1 vs. 93.0 ± 17.0 mmHg) and PPa (20.8 ± 4.8 vs. 34.0 ± 7.3 mmHg). Increased compliance by vasodilation decreased cPP (62.2 ± 20.2 vs. 45.2 ± 17.8 mmHg) without altering dP/dt, pPP or PPa. The emission coefficient changed with increasing cPP, but the reflection coefficient did not. These results agreed with *in silico* data obtained by independently changing contractility/compliance over the range observed *in vivo*.

**Conclusions:**

Ventricular contractility plays a key role in raising and amplifying PP, by altering aortic flow wave morphology.

## 1. Introduction

Hypertension, a leading cause of morbidity and mortality in the adult population (1), arises in large part from an increase in pulse pressure (2) and is a major risk factor for incident cardiovascular events particularly in older individuals (2). However, the haemodynamic basis of this increase is still debated. Traditionally, arterial stiffening and peripheral wave reflections have been considered the major determinants of the increase in PP and its amplification from the aorta to the periphery (where it is normally measured) (3–6). However, studies from Framingham have shown that peripheral wave reflections provide a relatively small contribution to age-related changes in central PP and augmentation pressure (7, 8). By contrast, recent studies have emphasised the potential importance of ventricular ejection dynamics, in combination with arterial stiffening, in determining central and peripheral blood pressure (BP) during early systole (8–12). In particular, left ventricular (LV) contractility, measured as the rate of increase in central BP during early systole (13), has been identified as a main determinant of aortic flow wave morphology, which in turn is a major determinant of PP (14, 15) and PP amplification (10, 16, 17). Quantification of the relative role of LV contractility and arterial stiffening in increasing central PP and amplifying it in the periphery would help in understanding the aetiology, prevention, and treatment of systolic hypertension.

The purpose of the present study was to examine the contributions of LV contractility and arterial stiffness to variations in aortic flow wave morphology and increased PP and PP amplification, and describe the underlying hemodynamic mechanisms. Both *in vivo* and *in silico* data were used. *In vivo* data was obtained in normotensive and hypertensive subjects; in the former, normal physiology was perturbed using vasoactive drugs with divergent effects on the heart and arteries. *In silico* data was simulated using a state-of-the-art model of cardiac dynamics coupled to a distributed model of arterial blood flow that enabled simulation of independent increases in either ventricular contractility or arterial stiffness that cannot be achieved *in vivo*. We examined the effects on central PP (cPP) and peripheral PP (pPP), PP amplification (PPa), and aortic flow of varying LV contractility or arterial stiffness, and studied the role played by aortic and peripheral wave reflections in raising cPP. Results showed that ventricular contractility plays a key role in raising and amplifying PP with hypertension.

## 2. Methods

Previously acquired *in vivo* data, both invasive and non-invasive, was used to examine relationships between pressure and aortic flow (18, 19).

### 2.1. *In vivo* data: invasive cohort

Invasive *in vivo* data included measurements of intra-aortic pressure and digital artery pressure waveforms previously acquired during diagnostic angiography in 23 patients (age 62 ± 10 years, BP 129 ± 24/66 ± 9 mmHg, means ± SD; see Table S1 in Supplementary Material) (18). Patients with acute coronary syndromes, those with significant valvular disease and rhythm other than sinus rhythm, were excluded from the study. Intra-aortic (central) pressure was measured using a Millar high–fidelity pressure tipped catheter (Millar Instruments, Houston, TX; sampling rate was flat to greater than 100 Hz) positioned in the proximal aortic root. Digital artery (peripheral) pressure was acquired simultaneously from the digital artery using a servo–controlled finger pressure cuff (Finometer; Finapres Medical Systems, The Netherlands; sampling rate: 128 samples per second). It has been previously shown that digital artery waveforms obtained in this way are virtually identical to radial artery waveforms acquired by tonometry using the SphygmoCor system (20). Baseline measurements of both central and peripheral pressures were obtained over at least ten cardiac cycles and then ensemble averaged. Sublingual glyceryl trinitrate (GTN, 500 µg), a vasodilator with some action on ventricular dynamics, was then administered and further measurements were acquired 2 min after GTN. Measurements took approximately 1–2 min to record, so they were centred at closer to 3 min after GTN. If there were substantial changes in heart rate or systolic BP (>10 bpm or >10 mmHg, respectively), measurements were continued until heart rate and systolic BP were stable.

### 2.2. *In vivo* data: noninvasive cohorts

The noninvasive *in vivo* data included measurements of aortic flow and central and peripheral blood pressure in a group of normotensive healthy volunteers (*n* = 10, age 47 ± 8 years, BP 103 ± 15/66 ± 9 mmHg, means ± SD) and hypertensive subjects (*n* = 93, age 46 ± 16 years, BP 134 ± 22/88 ± 14 mmHg, means ± SD) (19). Characteristics of the normotensive and hypertensive cohorts are given in Table S1 (Supplementary Material). In the normotensive cohort, haemodynamic properties were modulated by the administration of pharmacological drugs with different inotropic and vasoactive properties: dobutamine (DB), a positive inotrope with some vasodilator actions (2.5, 5, and 7.5 µg/kg per minute; Hameln Pharmaceuticals, Gloucester, United Kingdom), and noradrenaline (NA), a vasoconstrictor with some inotropic actions (12.5, 25, and 50 ng/kg per minute; Aguettant, Bristol, United Kingdom). Each drug was given on a different occasion separated by at least 7 days, and the order was randomized.

In both cohorts, the carotid artery waveform was used as a surrogate for the aortic pressure waveform (21). Peripheral pressure was measured at the radial artery. Both radial and carotid pressure waveforms were obtained by applanation tonometry performed by an experienced operator using the SphygmoCor system (AtCor, Australia; sampling rate: 128 samples per second). Waveforms were obtained at rest in all subjects and during each dose of vasoactive drugs in the normotensive subjects. For each measurement, approximately ten cardiac cycles were obtained, and ensemble averaged. Waveforms that did not meet the in–built quality control criteria in the SphygmoCor system were rejected. Brachial BP was measured in triplicate by a validated oscillometric method (Omron 705CP, Omron Health Care, Japan) immediately before measurements of tonometry and used to calibrate radial waveforms, and thus to obtain a mean arterial pressure (MAP) through integration of the radial waveform. Carotid waveforms were calibrated from MAP and diastolic brachial blood pressures (DBP) on the assumption of equality between proximal and peripheral DBP (22). Ultrasound imaging was performed by an experienced operator using a Vivid–7 ultrasound platform (General Electric Healthcare, UK). This provided a measurement of the flow velocity above the aortic valve using pulsed wave Doppler obtained from an apical five–chamber view. Flow velocity was extracted from the envelope of the spectrum, filtered to reduce speckles in late systole and early diastole, and averaged over at least three cardiac cycles.

### 2.3. *In silico* data: computational haemodynamics model

We used a previously described computational model of blood flow in the 116 largest human arteries of the head, thorax, and limbs (including the digital arteries in the hand) (23, 24). The model includes a state–of–the–art, lumped–parameter cardiac contraction model (25), representing the left side of the heart. The filling and contraction of the heart chambers are described by a time–varying elastance function relating the blood pressure and volume of the chambers and accounting for the strength and duration of the contraction and relaxation phases of myocardial activity in the left atrium and ventricle. The inflow to the cardiac model is the time–varying pulmonary venous flow rate entering the left atrium. Each artery of the network is characterized by its length, diameter, wall thickness, arterial wall stiffness, and arterial wall viscosity (24). All the peripheral branches are coupled to three–element Windkessel models that represent the resistance and compliance of the distal microvasculature.

The model parameters are representative of healthy subjects and can be defined for different age groups, from 25 to 75 years old (23). For the purpose of this study, the 45–year–old baseline subject was used to simulate blood flow and pressure at the aortic root and peripheral blood pressure at the radial artery. This model has age-specific mean values for all cardiovascular properties, and approximately matches the mean age of the normotensive and hypertensive cohorts (see Table S1, Supplementary Material). Cardiac or vascular parameters were changed independently to obtain hemodynamic properties spanning the range of values measured in the *in vivo* normotensive cohort. To simulate the vasoactive effects of NA and GTN, and to a lesser extent of DB, arterial compliance was modified by changing either geometrical or mechanical vascular parameters of the 45–year–old baseline subject, namely arterial stiffness (i.e., wall thickness and Young’s moduli) or luminal diameters, spanning the range of age-specific mean values from the 25– to the 75–year–old baseline subjects (23). To simulate the inotropic action of DB, and to a lesser extent of NA and GTN, left ventricular contractility in the baseline subject was increased by changing the parameters of the heart model. Based on our previous analysis of the sensitivity of simulated central blood pressure to cardiac parameters (26), the following parameters were varied: (i) the stroke volume within the corresponding values measured *in vivo* (see Table S2, Supplementary Material); and (ii) either the time of the left ventricular relaxation phase or the maximum amplitude of the contraction phase of the ventricular elastance function to produce the range of contractility index values measured *in vivo* (Supplementary Table S2).

### 2.4. Waveform postprocessing

For all *in vivo* and *in silico* measurements, cPP, pPP and PPa, obtained as the difference between the peripheral systolic blood pressure (pSBP) and the first systolic shoulder in central pressure (P1) (18) with the assumption of equal DBP, were analysed. Arterial stiffness was measured by arterial compliance (inversely related to stiffness) calculated as the ratio of stroke volume to cPP (27). Left–ventricular contractility was measured by the systolic index of contractility (28), which is calculated as the maximum rate of increase in early systolic central BP with respect to time (dP/dt) (13). Traditional wave separations analysis (29) was used to obtain forward (P_f_) and backward (P_b_) pressure components of the central pulse pressure wave, so that P_f_ + P_b_ = P−P_d_ with P the total blood pressure wave and P_d_ the diastolic blood pressure. Peripheral wave reflections were quantified by the peak reflection coefficient, $RC_{\;peak}$, calculated as the ratio of the peak value of P_b_ to that of P_f_. The amount of BP “emitted” at the aortic root towards downstream vessels relative to the amount of BP reaching the aortic root from downstream vessels was calculated using the peak emission coefficient, $\gamma_{\;peak}$, calculated as the ratio of the peak value of P_f_ to that of P_b_ (15). All simulations and postprocessing calculations were performed using customised Matlab software (The MathWorks, MA).

### 2.5. Statistics

Subject characteristics and results are presented as means ± SD. The effect of administering pharmacological drugs on haemodynamic measures was examined using paired *t*-tests. Baseline haemodynamic measures were compared with those measured at the maximum drug dose of GTN for the invasive cohort, and DB and NA for the normotensive cohort, and *p* < 0.05 was taken as significant. Correlation analyses were performed considering Pearson’s (R) and Spearman’s (r_s_) correlation coefficients. Pearson correlation evaluates the linear relationship between two continuous variables, whereas Spearman correlation evaluates their monotonic relationship (linear or not).

## 3. Results

### 3.1. Cardiac contractility, arterial compliance, and PP

**Figure 1:**
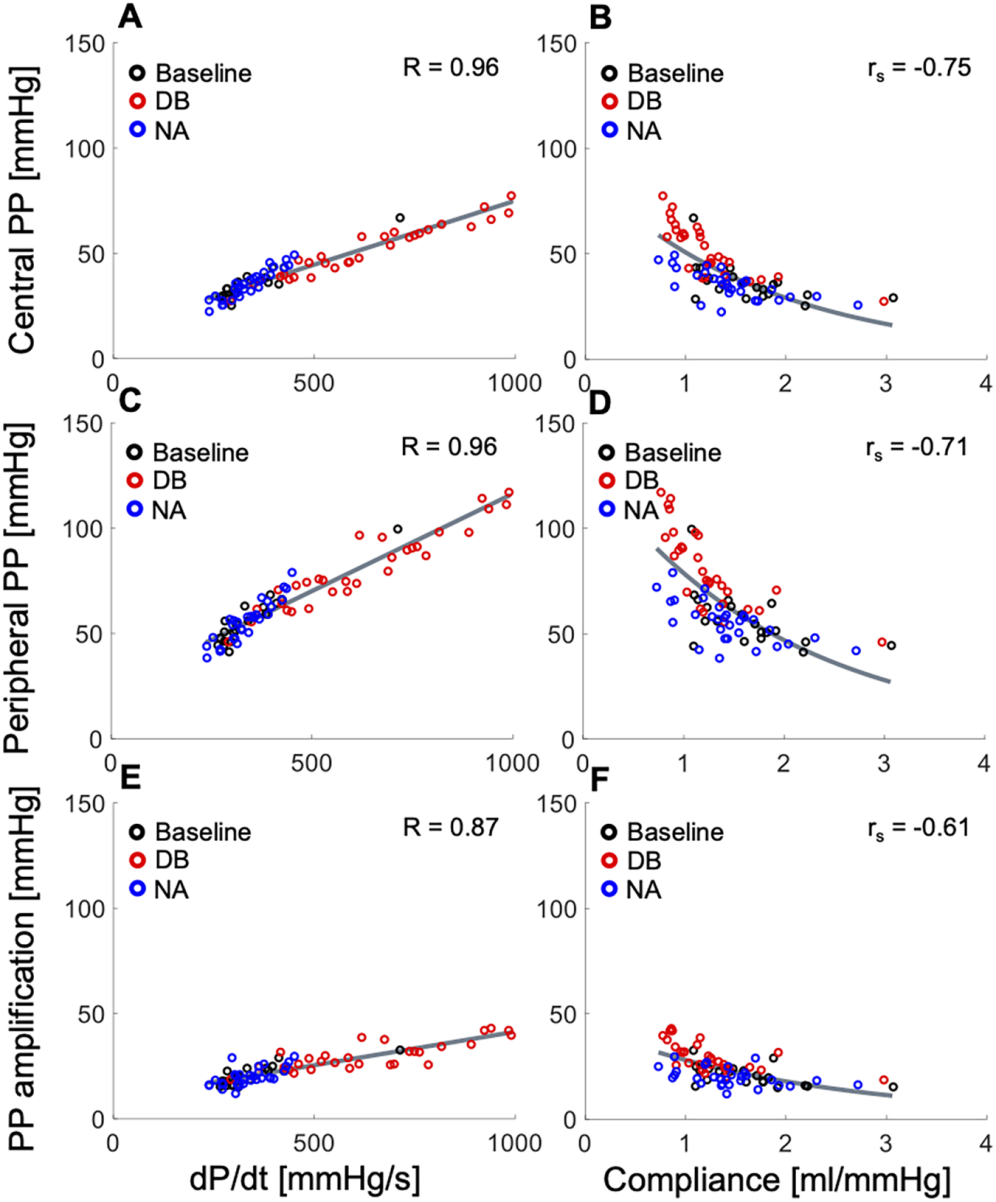
*In vivo* data showing relationships between the systolic index of contractility (dP/dt, left panels) or arterial compliance (right panels) and (top) central pulse pressure (PP), (middle) peripheral PP, and (bottom) PP amplification in the normotensive cohort receiving rising dose infusions of dobutamine (DB) and noradrenaline (NA) (see text for details). Pearson correlation coefficients (R) are provided for dP/dt and Spearman correlation coefficients (r_s_) are given for compliance. For a better interpretation of the figure, the reader is referred to the coloured web version of this article.

Both cPP and pPP were moderately to strongly associated with dP/dt for all the *in vivo* data (with Pearson’s correlation coefficient R = 0.96 for the normotensive cohort, Figures 1A,C; R > 0.77 and 0.76 for the hypertensive and invasive cohorts, respectively). In addition, both pulse pressures were inversely and nonlinearly associated with arterial compliance for the normotensive (Figures 1B,D) and hypertensive cohorts, although these associations were weaker than the corresponding associations with dP/dt (with Spearman’s correlation coefficients r_s_ < −0.71 and −0.45, respectively). For all *in vivo* cohorts, PPa was moderately to strongly associated with dP/dt (R = 0.87 for the normotensive cohort, Figure 1E; R = 0.81, and 0.70 for hypertensive and invasive cohorts, respectively) and showed a moderate to weak inverse correlation with arterial compliance (r_s_ = −0.61 for the normotensive cohort, Figure 1F; r_s_ = −0.30 for the hypertensive cohort). The correlations were the highest (R > 0.87 and r_s_ < −0.61) for the measurements in normotensive subjects, in whom haemodynamics were perturbed and therefore the range of variation in dP/dt and compliance was the greatest. The correlations between PP and dP/dt, and PP and compliance were not confounded by a correlation between dP/dt and compliance (R = −0.16 and −0.31 in the normotensive and hypertensive cohorts, respectively). Figures S1, S2 in the Supplementary Material show the correlation analyses for the hypertensive and invasive cohorts, respectively.

Administration of dobutamine significantly increased dP/dt (349.9 ± 101.2 vs. 754.0 ± 186.3 mmHg/s, *p* < 0.001) and led to larger cPP (36.1 ± 8.8 vs. 59.0 ± 10.8 mmHg), pPP (56.9 ± 13.1 vs. 93.0 ± 17.0 mmHg) and PPa (20.8 ± 4.8 vs. 34.0 ± 7.3 mmHg) (*p* < 0.001 each) in the normotensive cohort. In contrast, no significant changes in dP/dt, cPP, pPP and PPa were observed with administration of noradrenaline. Arterial compliance was found to be significantly decreased by dobutamine (1.63 ± 0.49 vs. 1.03 ± 0.22 ml/mmHg, *p* < 0.001) and, to a smaller amount, by noradrenaline (1.63 ± 0.49 vs. 1.29 ± 0.36 ml/mmHg, *p* = 0.04). In the invasive cohort, administration of glyceryl trinitrate significantly decreased cPP (62.2 ± 20.2 vs. 45.2 ± 17.8 mmHg, *p* = 0.004) and increased the time constant of the exponential decay of BP in diastole (0.47 ± 0.19 vs. 0.82 ± 0.68 s, *p* = 0.02), which depends on arterial stiffness (27), but did not affect dP/dt, pPP and PPa. Tables S2 and S3 in Supplementary Material show these haemodynamic measures for the normotensive and invasive cohorts, respectively, at baseline and after administration of pharmacological drugs.

**Figure 2:**
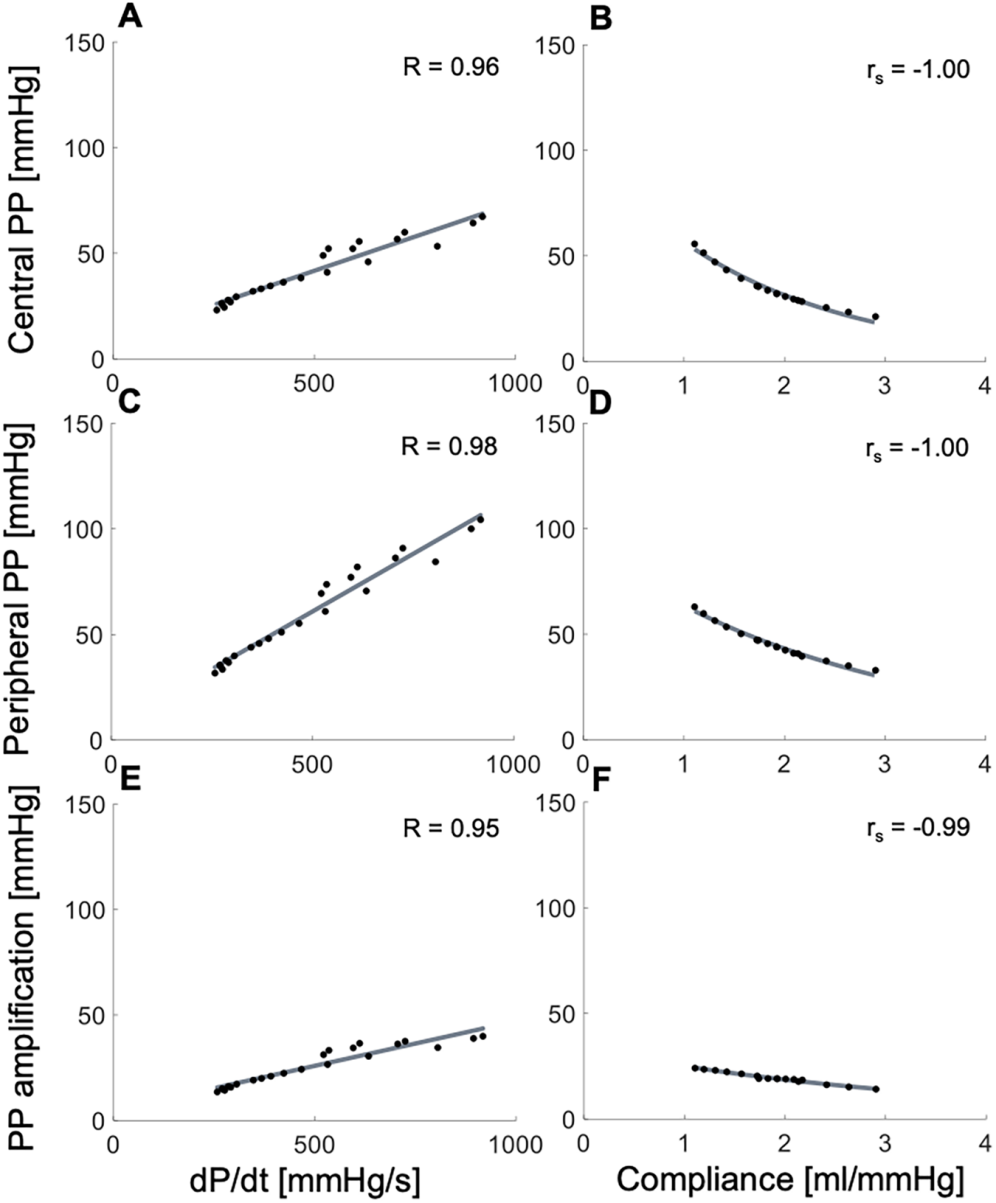
*In silico* data showing relationships between the systolic index of contractility (dP/dt, left panels) or arterial compliance (right panels) and (top) central pulse pressure (PP), (middle) peripheral PP, and (bottom) PP amplification. Pearson correlation coefficients (R) are provided for dP/dt and Spearman correlation coefficients (r_s_) are given for compliance.

*In silico*, variations of dP/dt, with compliance held constant, led to strong direct associations with cPP, pPP and PPa (Figures 2A,C,E), whereas changes in arterial compliance, with dP/dt held constant, produced strong, inverse, and nonlinear associations with cPP, pPP and PPa (Figures 2B,D,F). The range of variability in dP/dt and compliance, as well as the correspondent variations in PPs and PPa were consistent with those observed *in vivo* in Figure 1 and Figures S1, S2 (Supplementary Material).

### 3.2. Changes in aortic flow wave morphology

**Figure 3:**
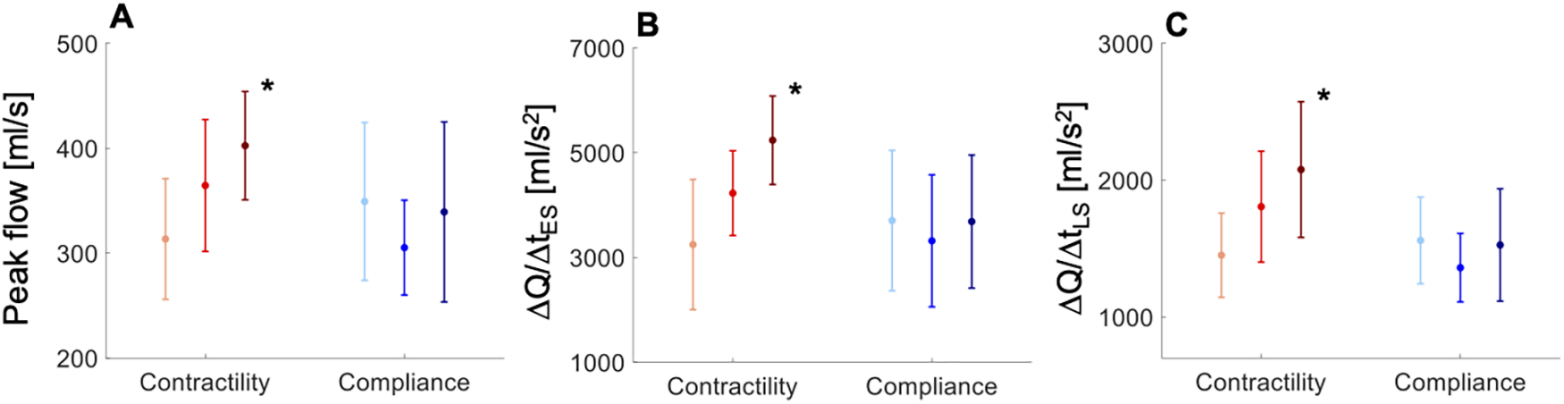
*In vivo* data showing variations in (**A**) aortic peak flow (PF), (**B**) rate of increase in early-systolic aortic flow (ΔQ/Δt_ES_), and (**C**) rate of decrease in late-systolic aortic flow (ΔQ/Δt_LS_) with increasing contractility (light-red line, dP/dt < 489 mmHg/s; red line, 489 < dP/dt < 740 mmHg/s; dark-red line, dP/dt > 740 mmHg/s) and compliance (light-blue line, C < 1.5 ml/mmHg; blue line, 1.5 < C < 2.3 ml/mmHg; dark-blue line, C > 2.3 ml/mmHg) in the normotensive cohort. All measures are shown as means ± SD. Asterisks indicate a significant difference between the first and third groups. For a better interpretation of the figure, the reader is referred to the coloured web version of this article.

*In vivo* flow data from the normotensive cohort showed an increase in peak aortic flow, and the rates of increase in early–systolic aortic flow and decrease in late–systolic aortic flow for increasing contractility, but no significant variation in these measures for increasing compliance (Figure 3). Administration of dobutamine in the normotensive cohort significantly increased peak aortic flow (323.9 ± 52.8 vs. 389.1 ± 65.1 ml/s, *p* = 0.015), the rate of increase in early–systolic aortic flow (3193.6 ± 793.0 vs. 4848.3 ± 450.4 ml/s^2^, *p* < 0.001), and the rate of decrease in late–systolic aortic flow (1433.6 ± 235.8 vs. 2020.0 ± 404.8 ml/s^2^, *p* = 0.001), without altering stroke volume (*p* = 0.78), whereas administration of noradrenaline did not affect these aortic flow measures (Table S2, Supplementary Material). Interestingly, these flow measures were greater in the hypertensive cohort than in the normotensive cohort at baseline: peak aortic flow (323.9 ± 52.8 vs. 353.3 ± 102.8 ml/s, *p* = 0.007), rate of increase in early–systolic aortic flow (3193.6 ± 793.0 vs. 3920.5 ± 1799.7 ml/s^2^, *p* < 0.001), and rate of decrease in late–systolic aortic flow (1433.6 ± 235.8 vs. 1543.2 ± 463.9 ml/s^2^, *p* = 0.01).

**Figure 4:**
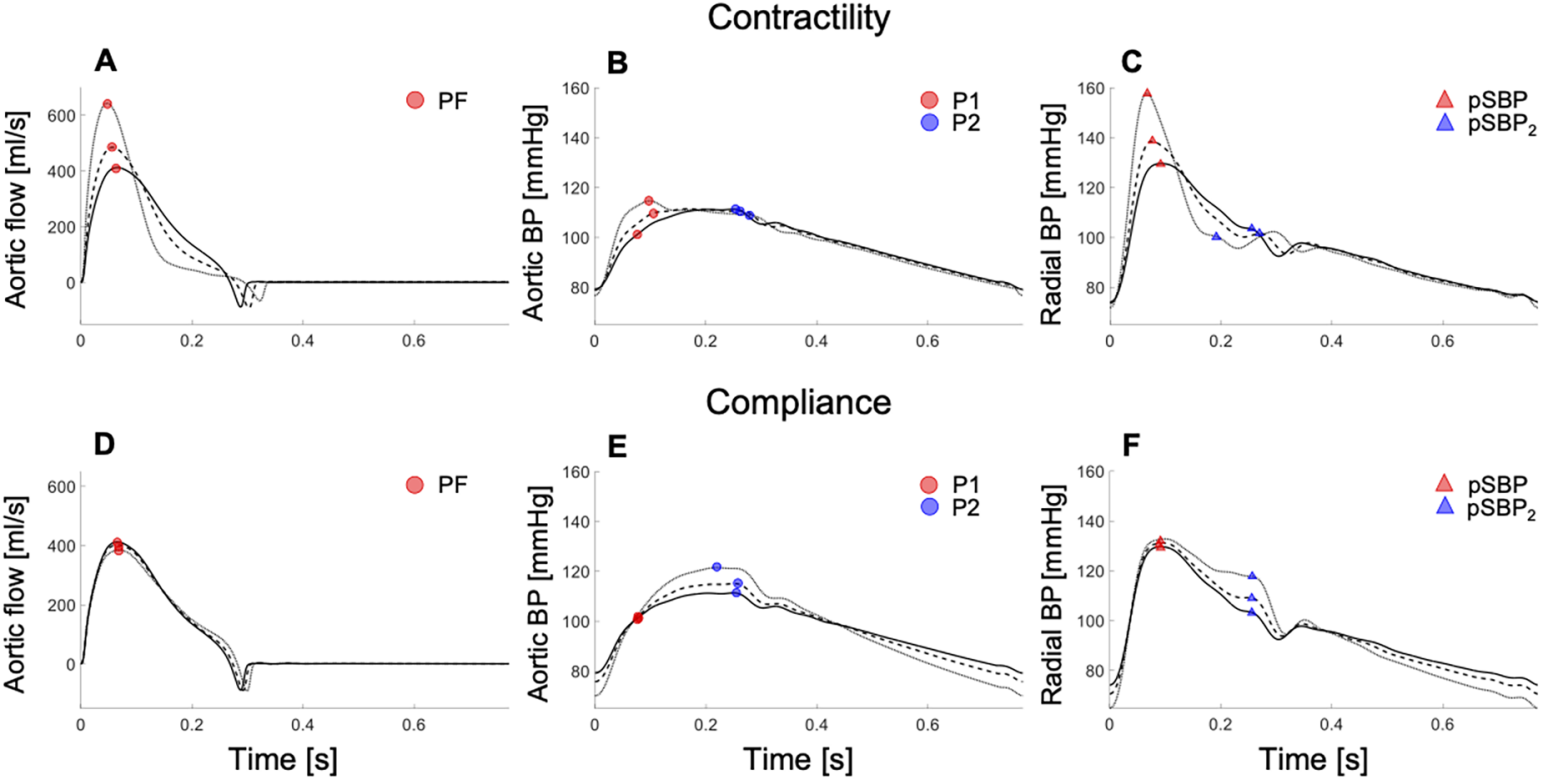
*In silico* data showing aortic flow (left), aortic pressure (centre), and radial pressure (right) waveforms with increasing cardiac contractility (top) and decreasing arterial compliance (bottom) in the 45–year–old virtual subject from baseline (solid lines; dashed and dotted lines indicated variations from baseline). Increasing contractility raised the peak aortic flow (PF) (**A**), first systolic shoulder in central pressure (P1) (**B**), and peripheral systolic blood pressure (pSBP) (**C**). Decreasing compliance increased the peak or second shoulder in central pressure (P2) (**E**) and the second peak or shoulder in the peripheral systolic blood pressure (pSBP_2_) (**F**), without affecting the peak aortic flow (**D**). For a better interpretation of the figure, the reader is referred to the coloured web version of this article.

*In silico* results agreed with *in vivo* results. Variations of dP/dt (by increasing the amplitude of the contraction phase in the LV elastance function and maintaining a constant stroke volume and compliance) increased peak aortic flow, and the rates of increase in early–systolic aortic flow and decrease in late–systolic aortic flow (Figure 4A). In contrast, little variation in aortic flow wave morphology was observed when compliance was changed with dP/dt held constant (Figure 4D).

### 3.3. Changes in blood pressure wave morphology

*In vivo* pressure data from the normotensive cohort showed an increase in the first systolic shoulder (P1) and, to a lesser extent, the second systolic shoulder (P2) in central pressure with increasing dose of dobutamine (101.9 ± 13.2 vs. 125.7 ± 7.3 mmHg, *p* < 0.01, and 98.8 ± 15.9 vs. 113.8 ± 12.9 mmHg, *p* = 0.03, respectively). On the other hand, increasing dose of noradrenaline predominantly raised P2, and, to a lesser extent, P1 (98.8 ± 15.9 vs. 121.9 ± 20.2 mmHg, *p* = 0.01, and 101.9 ± 13.2 vs. 115.5 ± 13.4, *p* = 0.03, respectively) (Table S2, Supplementary Material). Figure S3 (Supplementary Material) shows the effects of these pharmacological interventions on P1, P2 and central blood pressure wave morphology for a subject from the normotensive cohort. P1 and P2 were greater in the hypertensive cohort than in the normotensive cohort at baseline (127.8 ± 17.9 mmHg vs. 101.9 ± 13.2, *p* < 0.001, and 132.6 ± 24.0 vs. 98.8 ± 15.9 mmHg, *p* < 0.01, respectively).

*In silico*, independent changes in either contractility or compliance corroborated *in vivo* results. Increasing contractility, with compliance held constant, raised P1 and dP/dt in the aortic BP wave (Figure 4B), with little change in P2. Notably, P1 became the central systolic peak with high contractility, and then defined cPP, as observed *in vivo* (Supplementary Figure S3A). Decreasing compliance, with dP/dt held constant, did not affect central BP in early systole but led to an increase in P2 in the aortic BP wave (Figure 4E), in agreement with *in vivo* data (Supplementary Figure S3B). Similar changes in wave morphology were observed in the peripheral BP wave. Increasing contractility, with compliance held constant, raised the peripheral systolic BP (pSBP) and dP/dt (Figure 4C), whereas decreasing compliance, with dP/dt held constant, only affected the second peripheral systolic peak (pSBP_2_) (Figure 4F). pSBP and pSBP_2_ varied by +10% and −2%, respectively, when P1 was increased by +9%, and by +2% and +13%, respectively, when P2 changed by the same amount as P1 (Table S4, Supplementary Material). pSBP remained the peripheral pressure peak with variations in either contractility or compliance.

### 3.4. Relative contributions of contractility and compliance to PP

**Figure 5:**
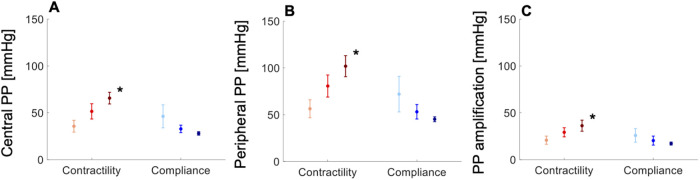
*In vivo* data showing variations in (**A**) central pulse pressure (PP), (**B**) peripheral PP, and (**C**) PP amplification with increasing contractility (light-red, dP/dt < 489 mmHg/s; red, 489 < dP/dt < 740 mmHg/s; dark-red, dP/dt > 740 mmHg/s) and compliance (light-blue, C < 1.5 ml/mmHg; blue, 1.5 < C < 2.3 ml/mmHg; dark-blue, C > 2.3 ml/mmHg) in the normotensive cohort. All measures are shown as means ± SD. Asterisks indicate a significant difference between the first and third groups. For a better interpretation of the figure, the reader is referred to the coloured web version of this article.

In the normotensive cohort, variations in cardiac contractility and arterial compliance within their respective physiological ranges led to greater contractility-driven increases in cPP (Figure 5A), pPP (Figure 5B) and PPa (Figure 5C) than compliance-driven decreases in the same measures. cPP, pPP and PPa increased at a rate of 46, 70 and 24 mmHg, respectively, per unit increase in contractility (Figure S4, Supplementary Material). These values were greater in the hypertensive cohort: 55, 70 and 31 mmHg, respectively, per unit increase in contractility (Supplementary Figure S5). *In silico*, these values were obtained with compliance held constant, which was not possible *in vivo* due to confounding factors. This led to comparable rates of increase in cPP, pPP and PPa than in the *in vivo* cohorts: 43, 73 and 28 mmHg, respectively, per unit increase in contractility (Supplementary Figure S6).

The rates of increase in cPP, pPP and PPa with compliance were compliance-dependent, increasing with decreasing compliance. In the normotensive cohort, the larger rates were 43, 63 and 21 mmHg, respectively, per unit decrease in compliance (Supplementary Figure S4). In the hypertensive cohort, these were 37, 29 and 10 mmHg, respectively, per unit decrease in compliance (Supplementary Figure S5). And, *in silico*, with dP/dt held constant, we obtained 35, 31 and 10, respectively, per unit decrease in compliance (Supplementary Figure S6).

### 3.5. The role of aortic and peripheral wave reflections

**Figure 6:**
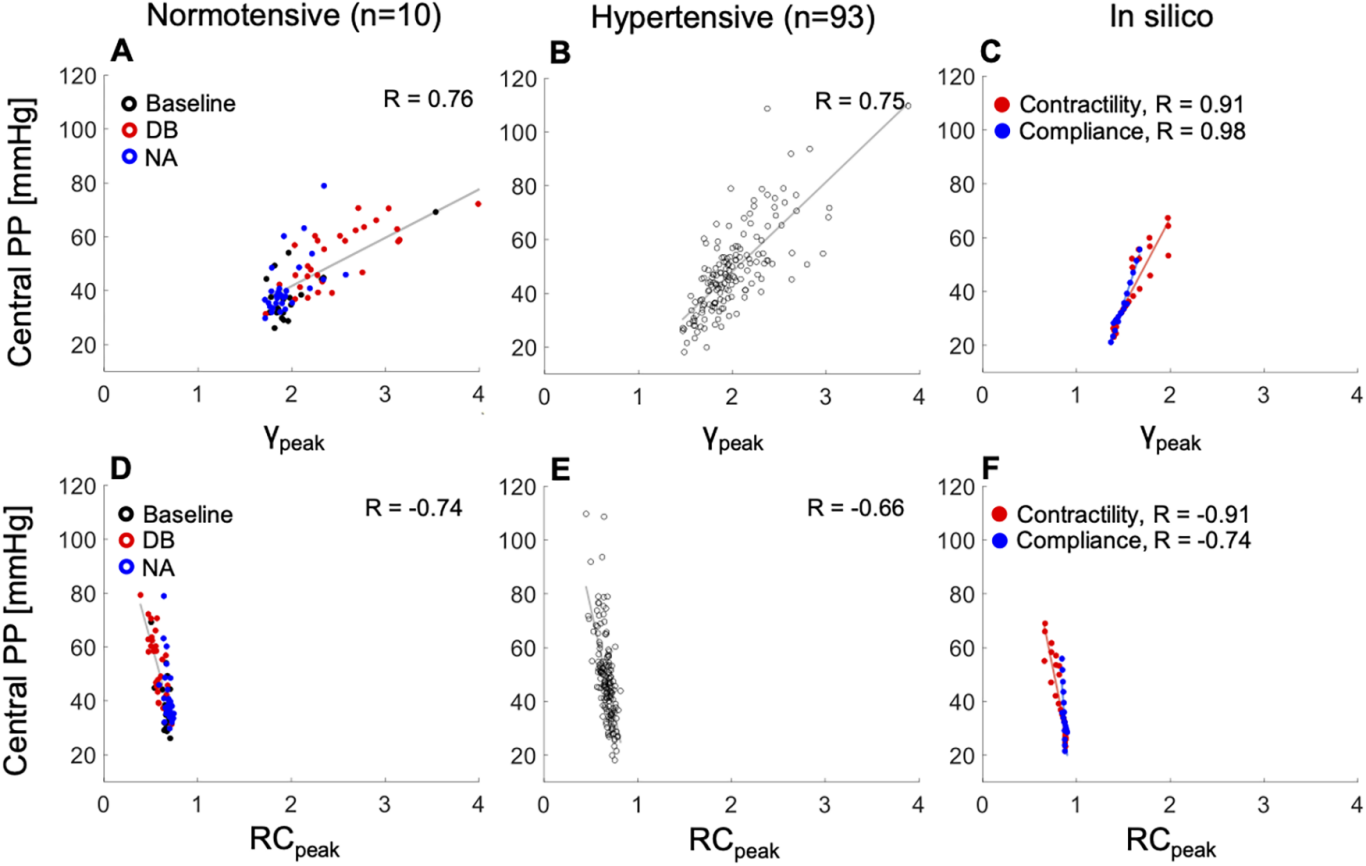
The relationship between peak emission ($\gamma_{\;peak}$, top) or reflection ($RC_{\;peak}$, bottom) coefficients and central pulse pressure (PP). Left: normotensive cohort receiving rising dose infusions of dobutamine (DB) and noradrenaline (NA). Centre: hypertensive cohort. Right: increasing cardiac contractility (red dots) or decreasing arterial compliance (blue dots) from baseline in the 45–year–old virtual subject. Pearson correlation coefficients (R) are provided. For a better interpretation of the figure, the reader is referred to the coloured web version of this article.

cPP was directly and strongly associated with a wide range of values of the peak emission coefficient at the aortic root, $\gamma_{\;peak}$, for the normotensive (R = 0.76) and hypertensive (R = 0.75) cohorts (Figures 6A,B), and showed a strong to moderate inverse correlation with a relatively narrower range of values of the peripheral wave reflection coefficient $RC_{\;peak}$, for the same two cohorts (Figures 6D,E; R = −0.74 and R = −0.66, respectively). *In silico*, cPP also increased with increasing $\gamma_{\;peak}\;$(Figure 6C) and decreasing $RC_{\;peak}$ (Figure 6F). Increasing contractility led to larger $\gamma_{\;peak}$ values than decreasing compliance, in agreement with the *in vivo* normotensive data: the increase in $\gamma_{\;peak}$ was significant with administration of DB, a mainly inotropic drug, and there was no significant change in $\gamma_{\;peak}$ with administration of NA, a mainly vasoactive drug (Figure 6A) (*p* < 0.001 vs. *p* = 0.5, Table S2, Supplementary Material). Furthermore, *in silico* $RC_{\;peak}$ decreased with increasing contractility and, to a much lesser extent, with variations in arterial compliance (Figure 6F), also in agreement with the normotensive data (Figure 6D): $RC_{\;peak}$ decreased more significantly with the administration of DB than NA (*p* < 0.001 vs. *p* = 0.03, Supplementary Table S2).

## 4. Discussion

Increased PP is the major hemodynamic change contributing to incident hypertension in ageing populations. It could result from arterial stiffening, increased peripheral wave reflections, or altered ventricular ejection dynamics. This study suggests that left ventricular contractility directly affects the aortic flow waveform which emerges as a main driver of the increase in PP and its amplification to the periphery, whereas arterial compliance does not alter aortic flow and has a relative smaller effect on PP and its amplification. Furthermore, pressure waves emitted at the aortic root, previously shown to be directly determined by aortic flow wave morphology (15), have a much greater contribution to the increase in PP with hypertension than pressure waves reflected from downstream to the aorta. Taken together, these results suggest that ventricular contractility and ejection dynamics play a key role in PP elevation and amplification with hypertension, in agreement with recent findings using only clinical data or models that did not account for ventricular–aortic coupling and had to resort to speculative assumptions on the underlying hemodynamic mechanisms (9–12). Our study used a complementary mix of *in vivo* and *in silico* data. *In silico* simulations allowed us to vary contractility and compliance over their *in vivo* pathophysiological ranges, in the absence of experimental errors, and in isolation, hence avoiding any *in vivo* confounding effects of compliance when varying contractility by the positive inotrope dobutamine or of contractility when varying compliance by the vasoconstrictor noradrenaline or the vasodilator glyceryl trinitrate. *In vivo* data measured in normotensive subjects, whose haemodynamics were altered by inotropic/vasoactive drugs, and hypertensive subjects, further strengthen the *in silico* results without resorting to modelling hypotheses.

Within the range of contractility and compliance values measured in the normotensive and hypertensive cohorts of the study, cPP, pPP, and PPa showed a greater variation with contractility than compliance. This is in agreement with findings from the Framingham Heart study which analysed data from 6,417 healthy subjects and showed that a smaller change in dP/dt than in compliance corresponded to an equal variation in cPP and pPP (67% vs. 90% variation in dP/dt and compliance, respectively, for 20 mmHg change in cPP and 18 mmHg change in pPP) (7). This study also showed values of peripheral DBP and SBP, and total compliance (64 ± 8 mmHg, 115 ± 11 mmHg, 1.7 ± 0.5 ml/mmHg, respectively), and it was possible to infer values of normotensive dP/dt (329 ± 83 mmHg/s), all in agreement with those in Table S1 in the Supplementary Material. Using the increases in cPP and pPP per unit dP/dt obtained from our *in silico* data, with compliance held constant, a 1 SD increase in dP/dt obtained from the Framingham Heart study leads to similar increases in pPP and cPP compared to a 1 SD decrease in compliance with *dP/dt* held constant (increases in cPP and pPP of +6 and +9 mmHg, respectively for *dP/dt* and of +9 and +8 mmHg, respectively for compliance). Therefore, at population level, the key role of contractility in raising and amplifying PP is corroborated. Moreover, the relationship between PPs and dP/dt showed in this work was confirmed by the ACCT study (30) conducted in a cohort of 4,001 healthy subjects, which indicated that cPP and pPP increase with ageing in parallel with an increase in dP/dt.

This study has identified distinct hemodynamic mechanisms underlying the increases in cPP, pPP and PPa with contractility and compliance. We start with the mechanisms of cPP increase. Changes in contractility alter the aortic flow waveform in early systole, producing noticeable changes in peak aortic flow, rate of increase in early–systolic aortic flow, and rate of decrease in late–systolic aortic flow, corroborating previous studies on the strong relation between systolic flow ejection and PP (14, 17). Alterations in the aortic flow wave in early systole have a direct effect on the aortic pressure wave via the water hammer equation [$\Delta P\;\;=\;\;Z_{C}\cdot \Delta Q$, with $Z_{C}$ the characteristic impedance depending on vascular properties only, and ΔP and ΔQ the changes in aortic pressure and flow in early systole (31)], before the arrival of downstream reflected waves. When vascular properties are unchanged, ΔP in early systole is directly proportional to ΔQ and a major contributor to systolic hypertension.

Changes in compliance do not produce the alterations in aortic flow wave morphology observed with varying contractility, although they can still affect the rate of increase in pressure in early systole (i.e., dP/dt) *via* changes in $Z_{C}$ through the water hammer equation. However, dP/dt did not vary significantly *in vivo* with administration of the vasoconstrictor noradrenaline or the vasodilator GTN. Instead, compliance produced changes in aortic pressure in late systole, when the BP wave can be described by a space-independent Windkessel model (14) and, hence, wave propagation phenomena is less relevant to explain increases in PP with decreases in compliance. Indeed, we found a greater variation in the amount of pressure waves emitted from the aortic root to downstream vessels with dobutamine-induced changes in contractility than with noradrenaline-induced changes in compliance. This result was also confirmed *in silico* with isolated changes in either contractility or compliance.

Variations in the amount of pressure waves emitted at the aortic root, quantified by the peak emission coefficient $\gamma_{\;peak}$, had a more predominant role in increasing cPP than variations in pressure waves reflected downstream the aorta, quantified by the reflection coefficient $RC_{\;peak}$. This result suggests a smaller contribution to increased cPP of peripheral wave reflections than wave activity occurring at the aortic root, in agreement with studies from Framingham showing a small contribution of peripheral wave reflections to age-related changes in cPP and augmentation pressure (7, 8).

We now focus on the mechanisms underlying changes in pPP and PPa. Increased contractility by administration of dobutamine raised both pPP and PPa, whereas increased/decreased compliance by administration of glyceryl trinitrate/ noradrenaline did not alter pPP or PPa. This finding suggests that contractility is the main driver for increased pPP and PPa, whereas compliance is a driver for increased cPP only. Contractility determines the first inflection point (P1) on the central blood pressure wave and the peripheral systolic blood pressure (pSBP) on the peripheral pressure wave, both occurring in early systole. Therefore, pPP—and consequently PPa—is determined by a wave propagation phenomenon initiated by a change in aortic flow wave morphology: the propagation of the early systolic raise in BP towards the periphery. This finding aligns with experimental (16) and theoretical (17) results. The latter study identified the rate of change of aortic flow with time in late systole (strongly correlated with ventricular ejection dynamics) as a main determinant of PPa, along with vessel radius and length from the aortic root to the periphery (17). It demonstrated that an increase in PPa occurs with a greater rate of decrease in aortic flow with time in late systole, which aligns with our *in vivo* and *in silico* findings. On the other hand, arterial compliance determines central and peripheral pressure peaks later in systole (P2 and pSBP_2_, respectively), with pSBP_2_ having a smaller magnitude than the contractility-dependent pSBP. As a result, compliance affects mainly cPP rather than pPP, in agreement with results using a central-to-peripheral transfer function (18). The association of compliance with cPP has been previously described by the Windkessel effect of central elastic arteries (14), where compliance undergoes a greater variation than in peripheral muscular arteries (higher smooth muscle content) (32, 33), consistent with physiological changes observed with ageing (23, 34).

This study is subject to several limitations. Carotid pressure is an imperfect surrogate of aortic pressure and subject to calibration errors. *In vivo* measurements of pressure and aortic flow velocity were not simultaneous and inevitably subject to experimental error that can propagate when calculating flow derived quantities, such as rate of increase in early–systolic aortic flow. However, these errors are likely to be random and unlikely to influence the conclusions of our study which have also been confirmed by a physics-based cardiovascular model. *In vivo*, it is challenging to alter LV contractility through pharmacological interventions without affecting other haemodynamic properties, including arterial compliance, and *vice-versa*. Dobutamine does not affect uniquely inotropy and may have some vasodilator actions affecting compliance, and noradrenaline and glyceryl trinitrate do not affect uniquely compliance and may have some inotropic actions. Glyceryl trinitrate may elicit some activation of the sympathetic nervous system (although this was not evidenced by an increase in heart rate). The *in silico* model was therefore used to address this limitation of *in vivo* data in determining the extent PPs, PPa, and aortic flow depend on properties of the heart and arterial tree, by varying the cardiac and vascular parameters in isolation and avoiding *in vivo* confounding factors. Furthermore, arterial compliance was estimated as the ratio of stroke volume to cPP. However, this method does not account for arterial outflow in systole and may, therefore, overestimate compliance (35). Finally, although our *in vivo* normotensive cohort was limited and included mainly middle-aged subjects, values of haemodynamic quantities obtained in the normotensive cohort were corroborated by other studies performed in larger cohorts.

## 5. Perspectives

The present results suggest that isolated systolic hypertension is more likely a result of dynamic (i.e., ventricular) than static (i.e., vascular) pathologies. Therefore, interventions that influence left ventricular contractility with a direct action on systolic ejection and aortic flow rate may be particularly effective in reducing PP and systolic hypertension, independent of vascular properties. Having established that peripheral systolic BP, which is used to assess clinical risk associated with hypertension and guide clinical care (18), is mainly determined by contractility also highlights the importance of targeting the ventricle when treating hypertension. Furthermore, having ascertained that PPa is mainly determined by contractility, and hence an indication of ventricular inotropy, noninvasive measurements of PPa from carotid (a surrogate for aortic pressure) to brachial or radial artery could offer cheap, pressure–based, assessment of left ventricular function.

## 6. Conclusions

By using a complementary mix of *in vivo* and *in silico* data we have shown that ventricular contractility influences systolic ejection, shaping aortic flow morphology and playing a primary role in raising and amplifying pulse pressure. Arterial compliance was found to play a secondary role and peripheral wave reflections to play a minor role. Targeting ventricular contractility may be important in preventing and treating systolic hypertension.

## Data Availability

The data analyzed in this study is subject to the following licenses/restrictions: The new in silico data supporting the conclusions of this article will be made available by the authors, without undue reservation. Requests to access these datasets should be directed to VC, valerio.caleffi@unife.it.
