# Supplementary material for: Cardiac contractility is a key factor in determining pulse pressure and its peripheral amplification
Source: Front Cardiovasc Med. 2023 Jun 23;10:1197842. doi: 10.3389/fcvm.2023.1197842 (PMC10326904; doi:10.3389/fcvm.2023.1197842)
Supplement: Supplementary file 1 [file Datasheet1.pdf]

Supplementary Material

Cardiac contractility is a key factor in determining pulse pressure and its peripheral amplification

Francesco Piccioli[1], Ye Li[2], Alessandro Valiani[1], Valerio Caleffi[1], Phil Chowienczyk[2], Jordi Alastruey[3*]

[1]Department of Engineering, University of Ferrara, Ferrara, Italy

[2]King's College London British Heart Foundation Centre, Department of Clinical Pharmacology, St Thomas' Hospital, London, United Kingdom

[3]Division of Imaging Sciences and Biomedical Engineering, King's College London, St. Thomas' Hospital, London, United Kingdom

*** Correspondence:**
Jordi Alastruey
jordi.alastruey-arimon@kcl.ac.uk

Table S1. Characteristics of the *in vivo* cohorts (at baseline) and *in silico* model.

**Characteristics**	**Normotensive cohort**	**Hypertensive cohort**	**Invasive cohort**	***In silico* model**
n	10	93	23	1
Age [year]	47±8	46±16	62±10	45
Sex [male %]	77	59	78	100
BMI [kg/m^2]	–	26.5±9.2	29.1±3.6	–
HR [bpm]	65±8	66±10	61±10	72
cSBP [mmHg]	103.2±15.4	134.4±22.3	129.3±23.6	103.7
pSBP [mmHg]	119.6±16.9	144.4±21.6	139.9±26	124.6
DBP [mmHg]	65.6±9.0	88.1±13.7	66.0±9.0	75.8
LVOT diameter [cm]	1.87±0.18	1.96±0.25	–	2.5
SV [ml]	59.9±13.0	77.9±24.8	–	66.4
dP/dt [mmHg/s]	349.9±101.2	421.9±112.4	343.2±114.3	347.4
C [ml/mmHg]	1.6±0.5	1.3±0.5	–	1.9

Values are numbers, percentage, or means ± SD. BMI, body mass index; HR, heart rate; cSBP, central systolic blood pressure; pSBP, peripheral systolic blood pressure; DBP, diastolic blood pressure; LVOT diameter, left ventricular outflow tract diameter; SV, stroke volume; dP/dt, contractility index, C, arterial compliance.

Table S2. Variations in haemodynamic measures with administration of pharmacological drugs in the normotensive cohort.

	Baseline	DB (max dose)	NA (max dose)
cPP [mmHg]	36.1 ± 8.8	59.0 ± 10.8<br>t(28) = -6.2, p < 0.001 *	36.8 ± 8.9<br>t(28) = -0.2, p = 0.85
pPP [mmHg]	56.9 ± 13.1	93.0 ± 17.0<br>t(28) = -6.4, p < 0.001 *	57.8 ± 12.4<br>t(28) = -0.2, p = 0.86
PP amplification [mmHg]	20.8 ± 4.8	34.0 ± 7.3<br>t(28) = -5.9, p < 0.001 *	21.0 ± 7.3<br>t(28) = -0.12, p = 0.90
dP/dt [mmHg/s]	349.9 ± 101.2	754.0 ± 186.3<br>t(12) = -6.4, p < 0.001 *	343.7 ± 69.7<br>t(25) = 0.2, p = 0.85
Compliance [ml/mmHg]	1.63 ± 0.49	1.03 ± 0.22<br>t(28) = 4.6, p < 0.001 *	1.29 ± 0.36<br>t(23) = 2.1, p = 0.04 *
Stroke volume [ml]	59.9 ± 13.0	61.1 ± 10.5<br>t(22) = -0.2, p = 0.78	56.02 ± 13.7<br>t(17) = 0.7, p = 0.45
Peak flow [ml/s]	323.9 ± 52.8	389.1 ± 65.1<br>t(15) = -2.7, p = 0.015 *	297.9 ± 67.3<br>t(15) = 1.1, p = 0.30
$\Delta Q/\Delta t_{ES}$ [ml/s^2]	3193.6 ± 793.0	4848.3 ± 450.4<br>t(22) = -6.8, p < 0.001 *	2807.0 ± 675.8<br>t(21) = 1.4, p = 0.18
$\Delta Q/\Delta t_{LS}$ [ml/s^2]	1433.6 ± 235.8	2020.0 ± 404.8<br>t(12) = -4.2, p = 0.001 *	1235.4 ± 288.4<br>t(16) = 1.9, p = 0.08
P1 [mmHg]	101.9 ± 13.2	125.7 ± 7.3<br>t(13) = -4.5, p < 0.001 *	115.5 ± 13.4<br>t(17) = -2.4, p = 0.03

P2 [mmHg]	98.8 ± 15.9	113.8 ± 12.9<br>t(17) = -2.3, p = 0.03	121.9 ± 20.2<br>t(17) = -2.7, p = 0.01 *
γ_{peak} [-]	2.01 ± 0.39	2.56 ± 0.66<br>t(47) = -3.7, p < 0.001 *	1.95 ± 0.21<br>t(26) = 0.7, p = 0.48
RC_{peak} [-]	0.66 ± 0.05	0.57 ± 0.07<br>t(47) = 4.9, p < 0.001 *	0.69 ± 0.03<br>t(31) = -2.3, p = 0.03 *
Proximal aorta diameter [cm]	1.9 ± 0.7	1.9 ± 0.8<br>t(16)=0.39; p = 0.70	1.9 ± 0.8<br>t(16) = 0.04; p = 0.96
Heart rate [Hz]	63 ± 10	73 ± 17<br>t(12) = -1.70; p = 0.11	53 ± 8<br>t(21)=2.96; p = 0.007 *

Haemodynamic measures of the normotensive cohort measured at baseline (second column), maximum dose of dobutamine (DB = 7.5 µg/kg per minute, third column), and maximum dose of noradrenaline (NA = 50 ng/kg per minute, fourth column). Haemodynamic measures are central pulse pressure (cPP), peripheral pulse pressure (pPP), pulse pressure (PP) amplification, contractility index (dP/dt), arterial compliance, stroke volume, peak aortic flow, rate of increase in early–systolic aortic flow ($\Delta Q/\Delta t_{ES}$), rate of decrease in late–systolic aortic flow ($\Delta Q/\Delta t_{LS}$), first systolic shoulder in central blood pressure wave (P1), second systolic shoulder in central blood pressure wave (P2), peak emission coefficient (γ_{peak}, see main article for details), peak reflection coefficient (RC_{peak}, see main article for details), proximal aorta diameter, and heart rate. Values are reported as mean ± SD. Two-sample t-tests were performed to compare the haemodynamic measures at baseline and with drug administration. Results are reported in the third and fourth columns as t(df) = t-value, where df stands for degrees of freedom from the t-test statistics. Asterisks indicate a significant difference between the haemodynamic variable at baseline and after maximum drug dose administration.

Table S3. Variations in haemodynamic measures with administration of GTN in the invasive cohort.

	**Baseline**	**GTN**
cPP [mmHg]	62.2 ± 20.2	45.2 ± 17.8<br>t(44) = 3.0, p = 0.004 *
pPP [mmHg]	73.1 ± 22.5	68.9 ± 17.9<br>t(44) = 0.7, p = 0.49
PP amplification [mmHg]	33.4 ± 16.3	30.3 ± 12.2<br>t(44) = 0.7, p = 0.48
dP/dt [mmHg/s]	378.8 ± 130.5	331.7 ± 117.5<br>t(44) = 1.3, p = 0.20
Time constant [s]	0.47 ± 0.19	0.82 ± 0.68<br>t(44) = -2.45, p = 0.02 *
Heart rate [Hz]	61 ± 10	63 ± 13<br>t(42) = -0.73, p = 0.46

Haemodynamic measures of the *in vivo* invasive cohort measured at baseline (second column) and after administration of glyceryl trinitrate (GTN, 500 µg; third column). Haemodynamic measures are central pulse pressure (cPP), peripheral pulse pressure (pPP), pulse pressure (PP) amplification, contractility index (dP/dt), time constant of the exponential relaxation decay [1] of the central blood pressure waveform, and heart rate. Values are reported as mean ± SD. Two-sample t-tests were performed to compare the haemodynamic measures at baseline and with GTN administration. Results are reported in the third column as t(df) = t-value, where df stands for degrees of freedom from the t-test statistics. Asterisks indicate a significant difference between the haemodynamic variable at baseline and after drug dose administration.

Table S4. Fiducial point variations.

Simulation	P1 (mmHg)	P2 (mmHg)	pSBP (mmHg)	$pSBP_2$ (mmHg)
Baseline	100	111	130	104
Increased contractility	110 (+9%)	112 (0%)	143 (+10%)	102 (-2%)
Decreased compliance	102 (+1%)	122 (+9%)	132 (+2%)	118 (+13%)

Fiducial points of the central and peripheral blood pressure (BP) waves measured in the 45–year–old virtual subject at baseline (first row), with increased contractility (second row) and with decreased compliance (third row). Percentage changes from baseline are shown in the second and third rows. P1 is the first inflection point of the central BP wave; P2 is the second systolic peak in the central BP wave; pSBP is the first systolic shoulder in the peripheral BP wave; $pSBP_2$ is the second peak or shoulder in the peripheral BP wave.

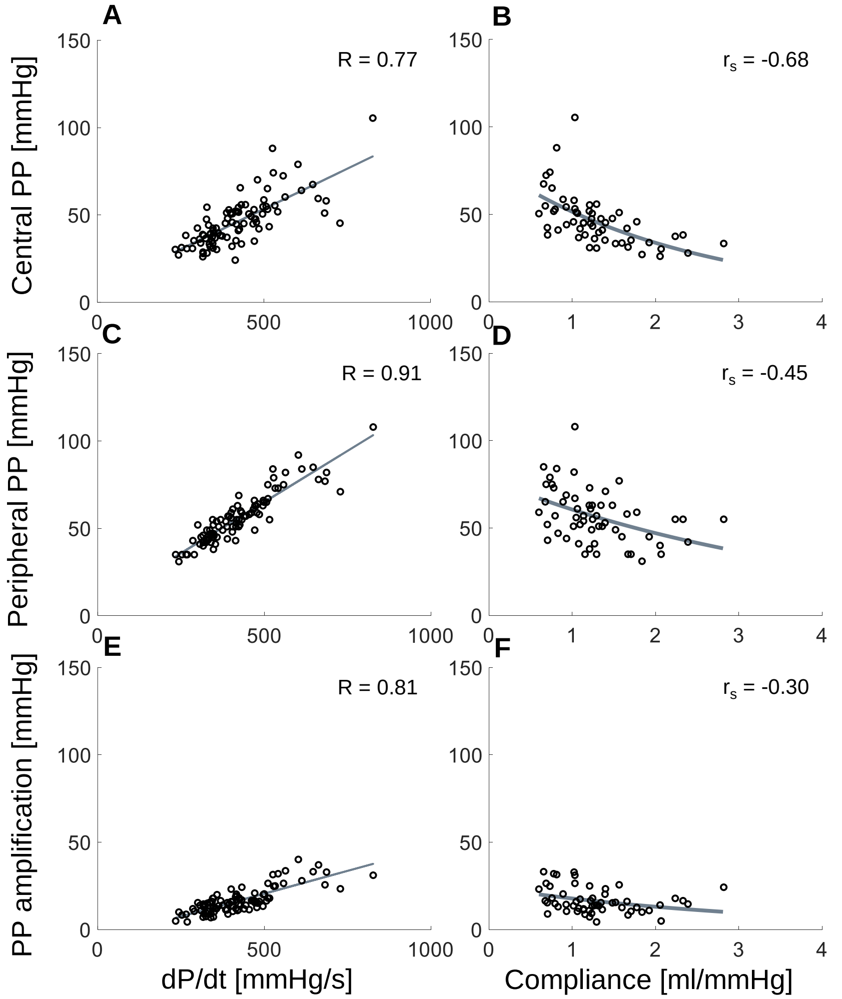

Figure S1. *In vivo* data showing relationships between the systolic index of contractility (dP/dt, left panels) or arterial compliance (right panels) and (top) central pulse pressure (PP), (middle) peripheral PP, and (bottom) PP amplification in the hypertensive cohort. Pearson correlation coefficients (R) are provided for dP/dt and Spearman correlation coefficients (r_s) are given for compliance.

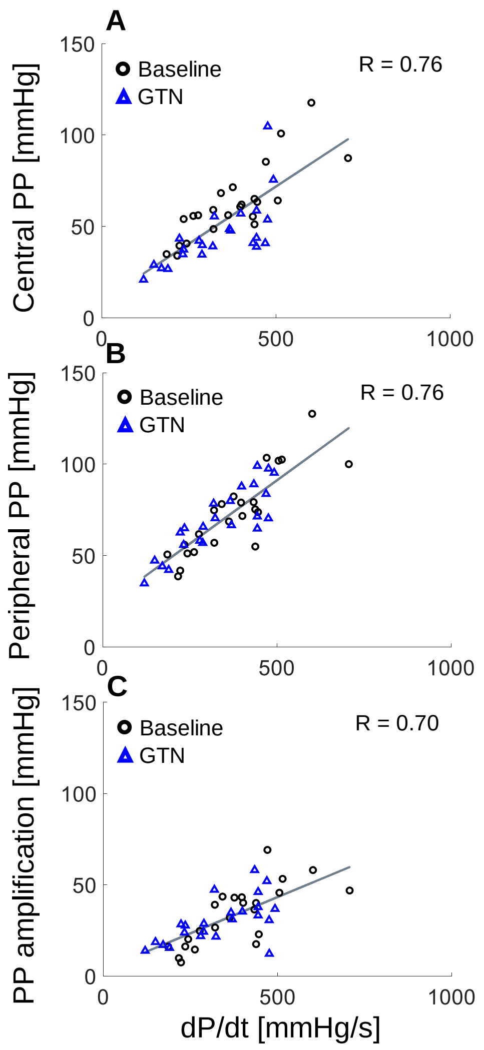

Figure S2. *In vivo* data showing the relationship between the systolic index of contractility (dP/dt) and (top) central pulse pressure (PP), (middle) peripheral PP, and (bottom) PP amplification in the invasive normotensive cohort receiving a dose infusion of glyceryl trinitrate (GTN). Pearson correlation coefficients (R) are provided for each plot.

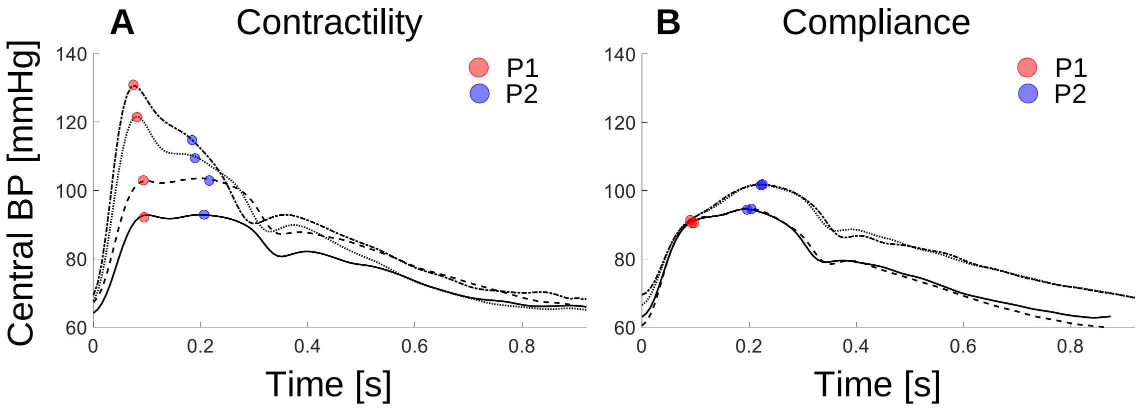

Figure S3. *In vivo* data showing variations in central blood pressure (BP) waveforms with administration of dobutamine (**A**, contractility) and noradrenaline (**B**, compliance) (baseline: solid line; dose 1: dashed line; dose 2: dotted line; dose 3: dash-dotted line) for a subject from the normotensive cohort. Increasing dose of dobutamine raised the first systolic shoulder (P1) and, to a lesser extent, the second systolic shoulder (P2) in central pressure. Increasing dose of noradrenaline predominantly raised peak or second shoulder in central pressure (P2) and, to a lesser extent, the first systolic shoulder (P1) in central pressure.

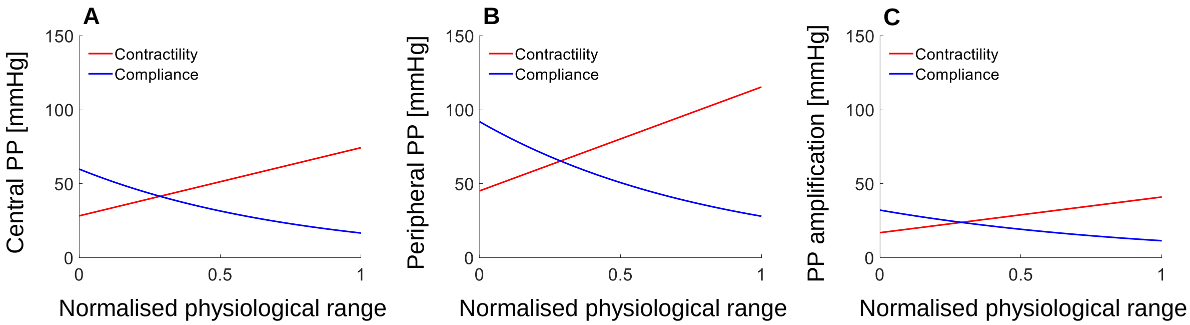

Figure S4. *In vivo* data showing variations in (**A**) central pulse pressure PP, (**B**) peripheral PP, and (**C**) PP amplification with normalised contractility (red) and compliance (blue) in the normotensive cohort. Trends calculated using the corresponding fitted curves in Figure 1 (main article). The normalised intervals reported in the x-axis were calculated using the minimum and maximum values of contractility and compliance observed in the cohort: from 230 to 990 mmHg/s for contractility and from 0.7 to 3.0 ml/mmHg for compliance.

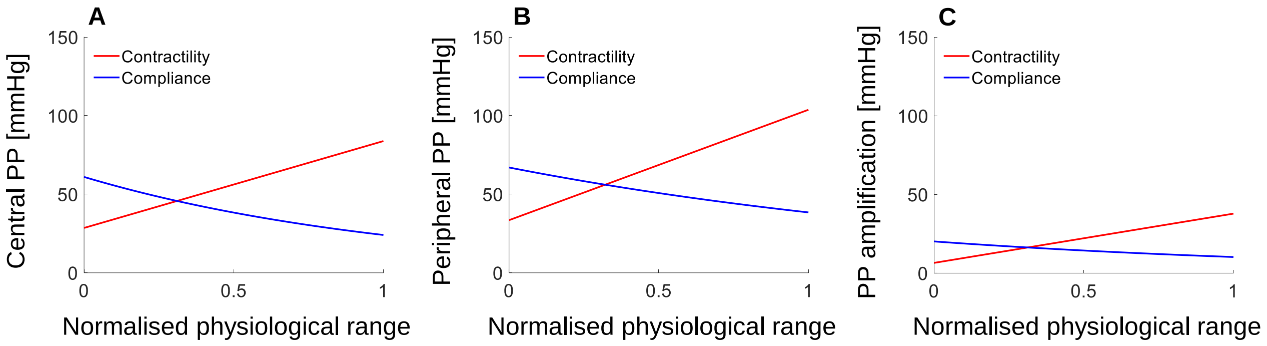

Figure S5. *In vivo* data showing variations in (**A**) central pulse pressure (PP), (**B**) peripheral PP, and (**C**) PP amplification with normalised contractility (red) and compliance (blue) in the hypertensive cohort. Trends calculated using the corresponding fitted curves in Figure S1. The normalised intervals reported on the x-axis were calculated using the minimum and maximum values of contractility and compliance observed in the cohort: from 230 to 830 mmHg/s for contractility and from 0.6 to 2.8 ml/mmHg for compliance.

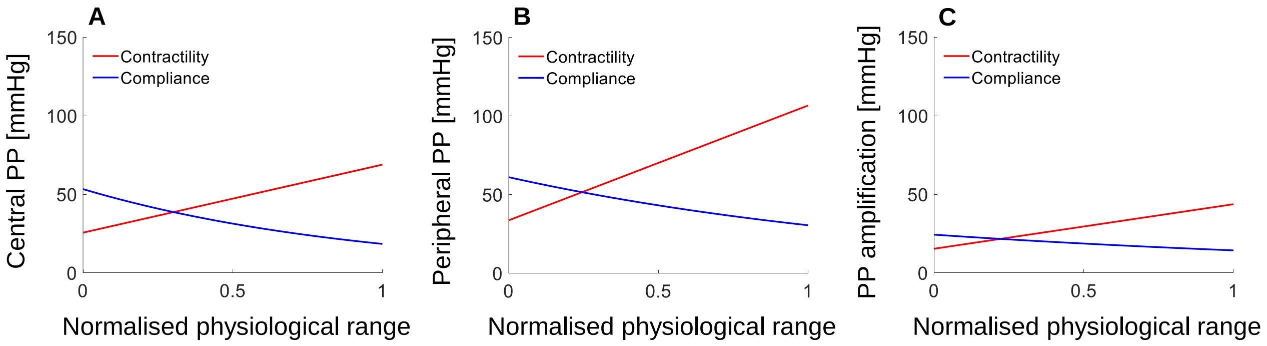

Figure S6. *In silico* data showing variations in (**A**) central pulse pressure (PP), (**B**) peripheral PP, and (**C**) PP amplification with normalised contractility (red) and compliance (blue). Trends calculated using the corresponding fitted curves in Figure 2 (main article). The normalised intervals reported on the x-axis were calculated using the maximum and minimum values of contractility and compliance observed in the *in silico* data: from 250 to 920 mmHg/s for contractility and from 1.1 to 2.9 ml/mmHg for compliance.

Reference

1. Mariscal-Harana J, Charlton PH, Vennin S, Aramburu J, Florkow MC, van Engelen A, Schneider T, de Bliek H, Ruijsink B, Valverde I, Beerbaum P, Grotenhuis H, Charakida M, Chowienczyk P, Sherwin SJ, et al. Estimating central blood pressure from aortic flow: development and assessment of algorithms. *American Journal of Physiology - Heart and Circulatory Physiology*. 2021;320(2):H494–H510. doi: 10.1152/AJPHEART.00241.2020
